# The effectiveness of a training program in improving the prenatal diagnosis accuracy of placenta accreta spectrum with ultrasound

**DOI:** 10.1002/ijgo.71127

**Published:** 2026-06-27

**Authors:** Nicha Vetvitayavatana, Pharuhas Chanprapaph, Julaporn Pooliam

**Affiliations:** ^1^ Division of Maternal‐Fetal Medicine, Department of Obstetrics and Gynaecology, Faculty of Medicine Siriraj Hospital Mahidol University Bangkok Thailand; ^2^ Clinical Epidemiology Unit, Faculty of Medicine Siriraj Hospital Mahidol University Bangkok Thailand

**Keywords:** placenta accreta spectrum, training program, ultrasound markers

## Abstract

**Objective:**

This study evaluates the effectiveness of a structured training program in improving prenatal ultrasound diagnostic accuracy for placenta accreta spectrum (PAS) and assesses trainees' ability to recognize individual ultrasound markers of PAS.

**Materials and Methods:**

This prospective educational study enrolled 16 third‐year obstetrics and gynecology residents who participated in a standardized PAS ultrasound training program. The program comprised a pre‐training assessment, a didactic lecture on PAS based on established ultrasound criteria, interactive case‐scenario exercises, and a 2‐month post‐training assessment. Diagnostic performance before and after training was evaluated using paired analyses, including overall diagnostic accuracy, sensitivity, and specificity for PAS. Recognition of individual grayscale and color Doppler ultrasound markers was also evaluated.

**Results:**

The training program significantly improved diagnostic performance. Overall diagnostic accuracy increased by 9.4% (from 65.6% to 75.0%). Diagnostic specificity improved significantly, by 17.5% (from 36.3% to 53.8%), while high sensitivity was maintained (95.0% vs. 96.3%). Improvements were also observed in the recognition of key PAS ultrasound markers. These findings are clinically significant, as increased specificity reduces false‐positive diagnoses and may decrease unnecessary referrals and healthcare resource utilization.

**Conclusion:**

A structured ultrasound training program significantly improves prenatal diagnostic accuracy for PAS and enhances recognition of key ultrasound markers among trainees. These findings support the implementation of standardized PAS ultrasound training and suggest potential benefit for broader adoption to improve prenatal detection of PAS.

## 1 INTRODUCTION

Placenta accreta spectrum (PAS) is defined as abnormal trophoblastic invasion into the myometrium due to a defect at the endometrial–myometrial interface.^1^, ^2^ It is one of the most serious causes of obstetric hemorrhage and peripartum hysterectomy, particularly when undiagnosed before delivery.^3^ The International Federation of Gynecology and Obstetrics (FIGO) classifies PAS into three grades according to the depth of placental invasion, reflecting increasing surgical complexity and maternal risk.^4^ Over recent decades, the global incidence of PAS has risen dramatically, largely driven by increasing cesarean delivery rates.^5^, ^6^ Earlier reports estimated an incidence of approximately one in 2500–4000 deliveries, whereas more contemporary population‐based studies report rates as high as one in 272 deliveries.^5^, ^6^ The risk of PAS increases proportionally with the number of prior cesarean deliveries and is further associated with placenta previa, advanced maternal age, assisted reproductive technologies, and prior uterine surgery.^4^, ^5^, ^6^, ^7^

Placenta accreta spectrum is associated with substantial maternal and neonatal morbidity, including massive hemorrhage, transfusion requirement, hysterectomy, adjacent organ injury, intensive care admission, preterm birth, and increased healthcare utilization.^2^, ^7^, ^8^, ^9^ Prenatal diagnosis is pivotal in improving outcomes. Planned delivery in tertiary referral centers with multidisciplinary expertise significantly reduces maternal blood loss, emergency hysterectomy, and peripartum complications.^7^, ^8^, ^9^, ^10^ However, despite improvements in imaging, 30%–50% of PAS cases remain undiagnosed antenatally.^11^, ^12^ In our tertiary referral center in Thailand, a retrospective review of 113 consecutive PAS cases demonstrated a prenatal detection rate of only 25.7%,^13^ highlighting a substantial gap between expected diagnostic performance and real‐world practice.

Ultrasonography is the first‐line imaging modality for PAS because of its accessibility, cost‐effectiveness, and real‐time diagnostic capability.^7^, ^14^, ^15^ A meta‐analysis of 3707 pregnancies demonstrated a sensitivity of 90.7% and specificity of 96.9% for ultrasound in diagnosing PAS,^16^ with performance comparable to magnetic resonance imaging in experienced hands. Nevertheless, ultrasound is inherently operator dependent, and diagnostic accuracy relies heavily on structured training, experience, and familiarity with standardized criteria.^17^ Grayscale and color Doppler markers (e.g., placental lacunae, loss of the clear zone, myometrial thinning, and abnormal uterovesical or subplacental vascularity) are widely used to support antenatal diagnosis.^3^, ^7^, ^17^ To improve consistency and communication, the European Working Group on Abnormally Invasive Placenta (EW‐AIP) proposed standardized ultrasound descriptors for PAS.^18^ Despite these efforts, variability in recognition and interpretation of these markers remains a major limitation in routine clinical practice.^18^, ^19^

Educational interventions have shown promise in enhancing PAS detection using still ultrasound images.^20^, ^21^ However, structured video‐based ultrasound training incorporating standardized PAS markers has not been adequately evaluated. Recent advances in medical research have highlighted the growing role of machine learning and deep learning in disease classification and medical image analysis. Applications include cardiovascular signal classification, cancer detection using imaging modalities, and broader clinical prediction models, all of which have demonstrated improved diagnostic accuracy through automated pattern recognition and feature extraction.^10^, ^22^, ^23^ However, in clinical fields such as obstetric ultrasound, diagnostic performance remains highly dependent on operator expertise, and the implementation of such technologies in routine practice remains limited, particularly in resource‐constrained settings. Therefore, structured educational interventions aimed at improving human interpretation skills remain essential and complementary to technological advancements.

Given the rising incidence of PAS, the substantial consequences of missed prenatal diagnosis, and the demonstrated gap in detection within our institution, optimizing ultrasound training represents a critical and potentially high‐impact strategy to improve maternal outcomes. The present study aimed to evaluate the effectiveness of a structured, video‐based PAS ultrasound training program in improving diagnostic accuracy and recognition of individual PAS ultrasound markers among trainees.

## 2 MATERIALS AND METHODS

This prospective educational study was conducted in the Department of Obstetrics and Gynaecology in the Faculty of Medicine at Siriraj Hospital, Mahidol University, Bangkok, a large medical school and a renowned tertiary referral center for PAS cases nationwide.

A total of 16 third‐year obstetrics and gynecology residents were recruited into the study between April 2022 and June 2022. Participants were part of a standardized national residency training program, with prior structured exposure to obstetric ultrasound and maternal–fetal medicine (MFM), supporting homogeneity in baseline training. All participants completed the study, as the training program was conducted within a structured academic schedule, resulting in no loss to follow‐up. Eligible participants were third‐year obstetrics and gynecology residents who had completed a maternal–fetal medicine rotation, possessed basic obstetric ultrasound skills, and agreed to participate in the program. Participants who declined to participate in the program or did not complete the training course within the given time were excluded from the study. This study was approved by the Siriraj Institutional Review Board (no. SI 278/2022).

Sample size calculation was based on the study by Dimitrova et al.^20^ that reported a 17.3% increase in diagnosis accuracy of PAS after enrollment in the training program. If the test significance level was 0.01 (type I error = 0.01, two sided) and the power of the test was 99% (type II error = 0.01), a total of 10 participants was required. Assuming that 30% of data might be lost or incomplete during the process of the study, the author decided to recruit a total of 16 participants into the program.

### 2.1 Preparation of placenta accreta spectrum pretest, posttest, and placenta accreta spectrum core lecture

The resources for the preparation of the PAS pretest, PAS posttest, and PAS core lecture were derived from specific case selection based on the final histopathological findings, the quality of ultrasound clips, and the obstetric outcomes. Prenatal ultrasound video clips of PAS and non‐PAS cases were obtained from the hospital digital database. The selected ultrasound video clips were then reviewed and validated by three MFM experts in our maternal and fetal medicine unit for content validity. Five PAS video clips were chosen if they met the usual optimization requirements for ultrasound video clips (sufficient bladder filling, proper probe pressure, and complete obtainment of the sagittal plane of the placental bed uterovesical area and adjacent organs) and had histopathologically verified evidence. Although this study does not involve machine learning models where ablation studies and cross‐validation are typically applied, methodological rigor was ensured through expert validation of cases, use of standardized ultrasound markers based on established guidelines, and consistent use of the same dataset in both pretest and posttest assessments with randomized case ordering to reduce recall bias. These approaches were implemented to enhance internal validity and minimize overfitting of participant responses to specific case sequences. Among five cases of PAS, two cases were PAS grade I and three cases were PAS grades II–III. Another five non‐PAS video clips were selected if they shared certain ultrasound characteristics with PAS, such as placenta previa or adenomyosis with hypervascularized myometrium and had proven evidence that the placenta could be completely removed at the time of delivery. For the pretest and posttest assessment, five cases of PAS and five cases of non‐PAS were selected, but they were arranged in different order. Each case's video clip lasted for 4 min and featured three to four loops of the whole case. The content validity of the pre‐and posttest assessment as well as the test reliability were evaluated by standard protocol. The trainees were blinded to the proportion of PAS and non‐PAS cases in the exam. In the PAS core lecture, video clips for the pretest and posttest evaluation will not be used to reduce trainee recollection bias.

The flowchart of our study is shown in Figure 1. After written consent forms were completed, all the trainees were given an orientation about the program and were assigned for 45‐min pretest assessment. The trainees were assigned to describe each PAS ultrasonographic marker and provide the final diagnosis by ticking the appropriate box (yes or no) in a Google form (Figure 2). Then, trainees participated in a 40‐min onsite PAS core lecture. The training program was designed using a structured educational framework consisting of three main components: (i) theoretical knowledge acquisition, (ii) visual pattern recognition, and (iii) applied case‐based practice. The theoretical component included PAS pathophysiology and grading, as well as comparison between normal and abnormal placental findings. The visual recognition component focused on the identification of standardized ultrasound markers, including grayscale and color Doppler features based on established descriptors. The applied component involved interpretation of dynamic ultrasound video cases to simulate real clinical scenarios. The training also incorporated correlation between ultrasound findings and surgical or histopathological outcomes, along with technical guidance on image acquisition (e.g., probe positioning, probe pressure, and bladder filling). Interactive case scenarios were provided to reinforce learning (Figure 3). Participants were subsequently given access to recorded lecture materials (Siriraj e‐Learning and Education Community; SELECx) on a website for review over a 2‐month period (Figure 4).

**FIGURE 1:**
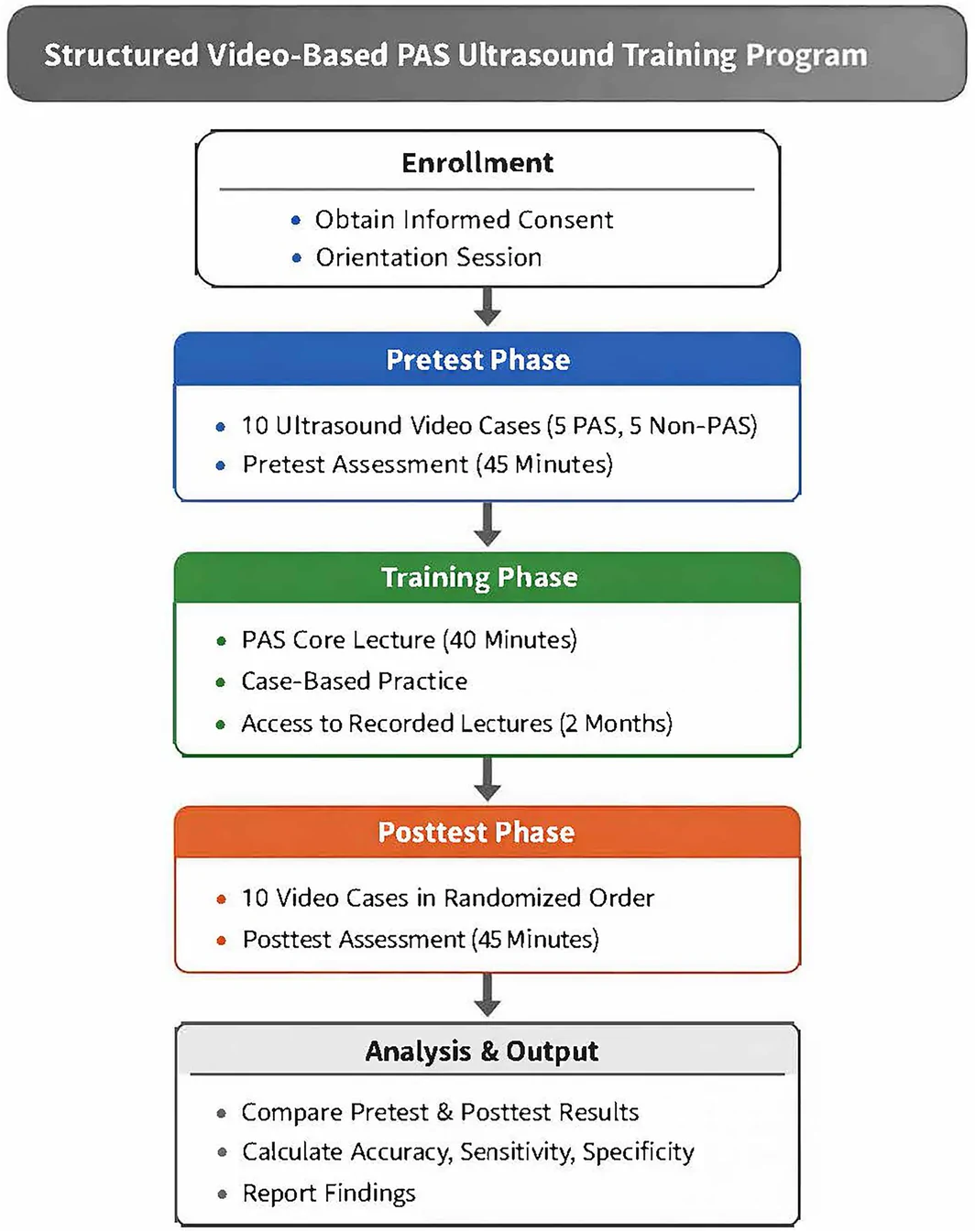
Study flowchart of participant enrollment, pre‐training assessment, training program, and post‐training assessment.

**FIGURE 2:**
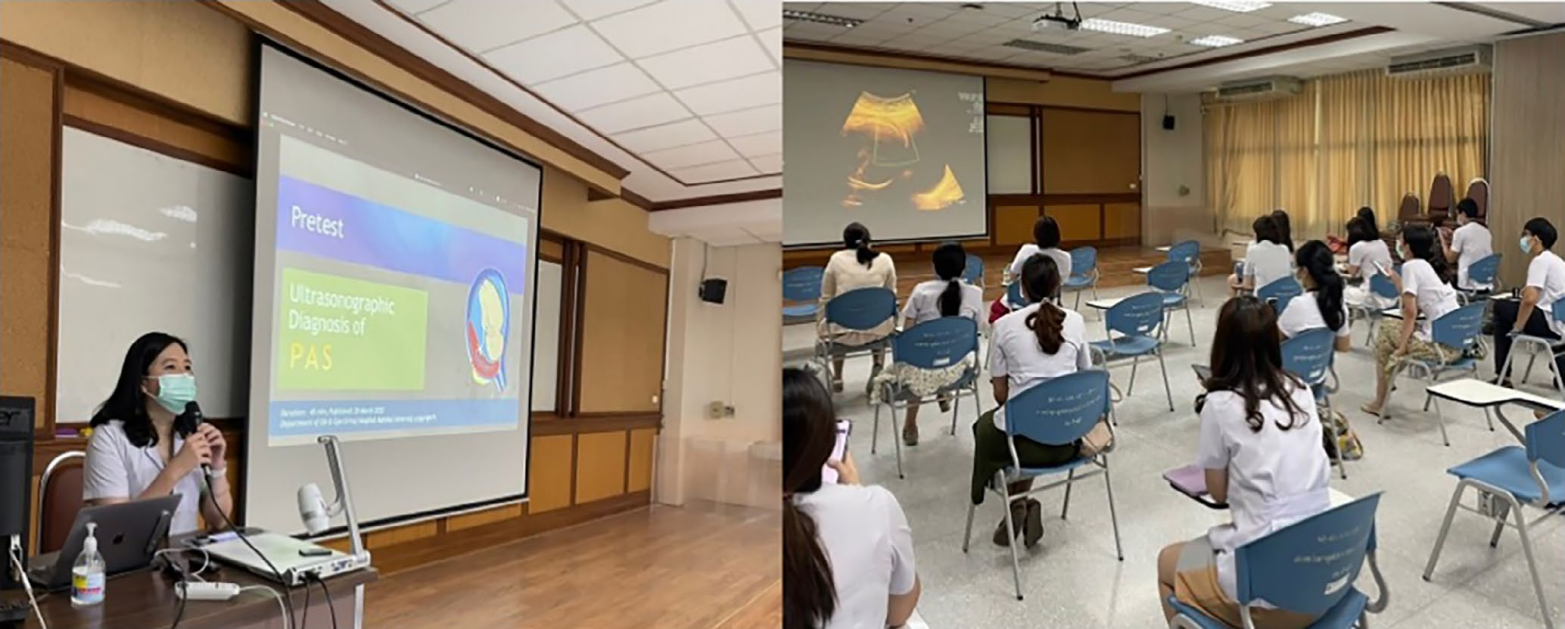
Pre‐ and post‐training placenta accreta spectrum (PAS) assessments. Trainees completed a 45‐min assessment evaluating 10 ultrasound video clips (five PAS and five non‐PAS cases) presented in random order. Participants identified individual PAS ultrasonographic markers and provided a final diagnosis using an online questionnaire. Assessments were conducted 2 months apart to minimize recall bias. Any additional individuals visible in the image were observers and not study participants.

**FIGURE 3:**
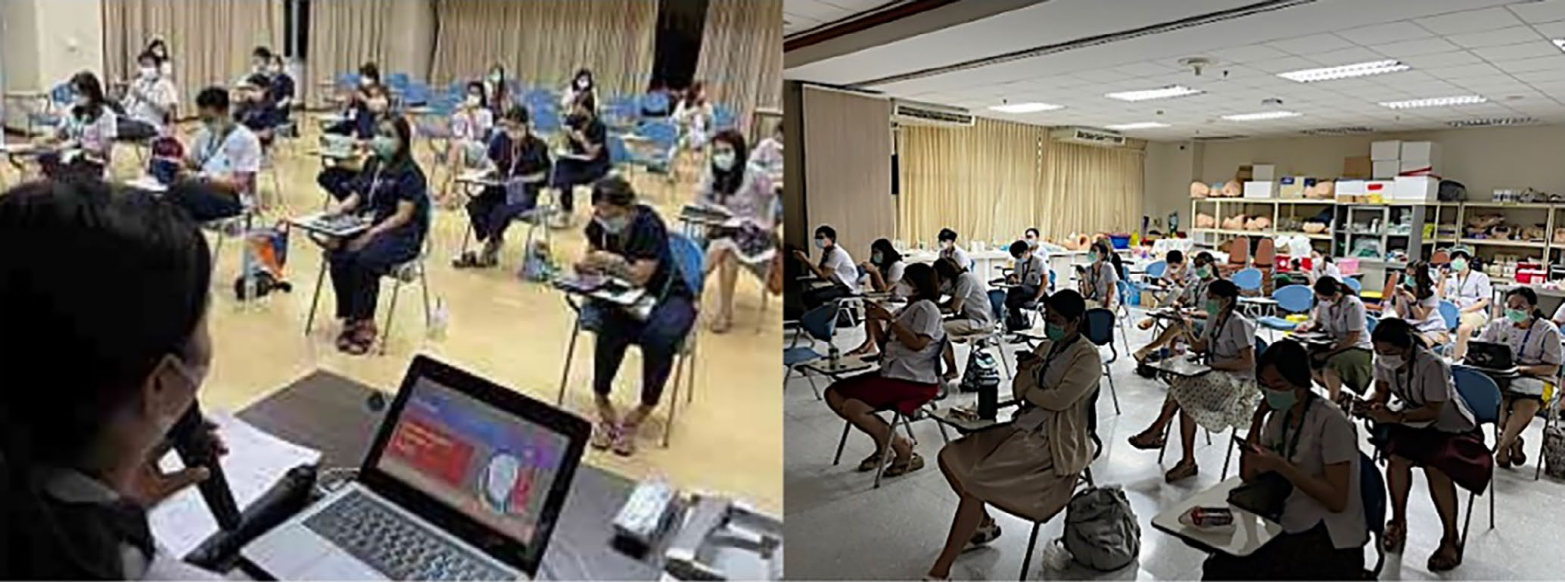
Onsite placenta accreta spectrum (PAS) core lecture. All trainees attended a 40‐min lecture covering PAS pathophysiology and grading, key ultrasonographic markers, and techniques for optimal image acquisition. Representative PAS video clips were demonstrated, followed by access to recorded lecture materials for self‐directed learning over a 2‐month period. Additional visible individuals were observers and not study participants.

**FIGURE 4:**
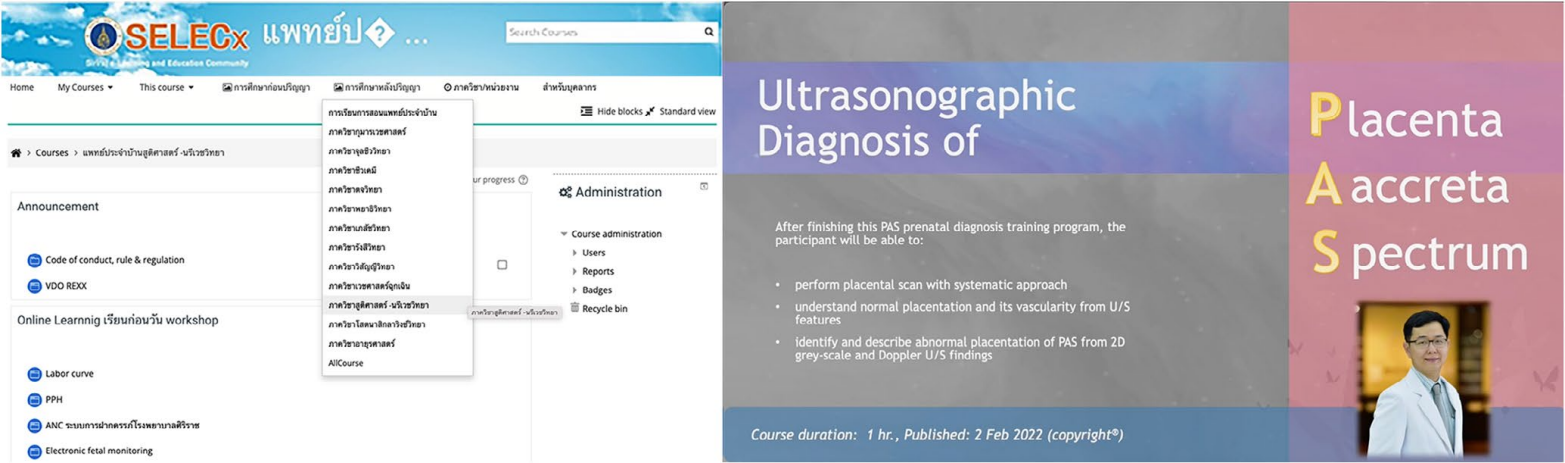
Online access to placenta accreta spectrum (PAS) lecture via the Siriraj e‐Learning and Education Community (SELECx) platform. Following completion of the onsite lecture, trainees were granted access to the recorded lecture for self‐directed learning over a 2‐month period.

After training, a 45‐min posttest using the same 10 cases (reordered) was conducted to minimize recall bias. All responses were collected for statistical analysis. To enhance clarity, the study workflow is summarized in Supplementary Figure S1. The total structured training time required per participant was approximately 135 min, using existing institutional resources without additional hardware requirements.

### 2.2 Statistical analysis

All analyses were performed using SPSS Statistics 21. Descriptive statistics were used to describe baseline characteristics of the trainees, including age, years of medical practice, and years of transabdominal ultrasound experience, which were analyzed to account for prior experience. Continuous data was presented using the mean, standard deviation (SD), median, and range while categorical data was presented in amount and percentage. The dataset characteristics and statistical analysis parameters are summarized in Supplementary Table S1. For comparing the difference between PAS detection rate before and after the training program, a paired *t*‐test was used for normal distribution data and the Wilcoxon sign rank test was used for abnormal distribution data. The diagnostic performance before and after the training program was evaluated using accuracy, sensitivity, specificity, positive predictive value (PPV), and negative predictive value (NPV), with reporting of 95% confidence intervals.

## 3 RESULTS

A total of 16 obstetrics and gynecology residents were recruited into this study. All participated to the end of the training program without any single dropout. Baseline characteristics of the trainees are demonstrated in Table 1. The mean age of the trainees was 29.88 ± 0.6 years, with an average of 5.5 years of medical practice and 3 years of transabdominal ultrasound experience.

**TABLE 1:** Baseline characteristics of participants according to improvement in PAS detection skills.

Characteristic	Overall (*n* = 16)	Improved PAS detection (*n* = 12)	No improvement (*n* = 4)	*p*
Age (years), mean ± SD	29.9 ± 0.6	29.8 ± 0.5	30.3 ± 1.0	0.552
Sex, *n* (%)				0.529
Female	13 (81.2)	9 (75.0)	4 (100)	
Male	3 (18.8)	3 (25.0)	0 (0)	
Years of medical practice, median (range)	5.5 (5–6)	5.5 (5–6)	5.5 (5–6)	0.262
TAS experience (years), median (range)	3 (1–6)	4 (1–6)	3 (3–3)	0.262
Number of PAS lectures watched, median (range)	2 (1–5)	2 (1–5)	2 (2–3)	0.684
PAS cases encountered before enrollment, *n* (%)				0.537
0–3 cases	13 (81.3)	10 (83.3)	3 (75.0)	
≥ 4 cases	3 (18.7)	2 (16.7)	1 (25.0)	
PAS cases with TAS performed before enrollment, *n* (%)				0.862
0–3 cases	14 (87.5)	10 (83.3)	4 (100)	
≥ 4 cases	2 (12.5)	2 (16.7)	0 (0)	
Baseline PAS knowledge, *n* (%)				0.306
Average	9 (56.3)	7 (58.3)	2 (50.0)	
Fair	3 (18.7)	3 (25.0)	0 (0)	
Poor	4 (25.0)	2 (16.7)	2 (50.0)	
Knowledge of 2D grayscale PAS markers, *n* (%)				0.446
Good	2 (12.5)	1 (8.3)	1 (25.0)	
Average	9 (56.3)	6 (50.0)	3 (75.0)	
Fair	4 (25.0)	4 (33.3)	0 (0)	
Poor	1 (6.3)	1 (8.3)	0 (0)	
Knowledge of color Doppler PAS markers, *n* (%)				0.587
Average	9 (56.3)	6 (50.0)	3 (75.0)	
Fair	5 (31.3)	4 (33.3)	1 (25.0)	
Poor	2 (12.5)	2 (16.7)	0 (0)	

*Note*: Data are presented as mean ± SD, median (range), or number (%).

Abbreviations: 2D, two‐dimensional; PAS, placenta accreta spectrum; TAS, transabdominal ultrasound; U/S, ultrasound.

The overall diagnoses of 10 cases in the pre‐ and post‐test assessment made by trainees are shown in Table 2. Before training, the true positive diagnosis of PAS grade II–III reached 100%, reflecting that the trainees had no problems in making the diagnosis of a severe form of PAS. After training, there was an increase in true positive and true negative diagnosis of PAS cases, which indicated that the ability to distinguish a mild form of PAS (PAS grade I) from non‐PAS cases increased with training.

**TABLE 2:** Overall PAS diagnosis made by trainees before and after training.

Trainee's diagnosis performance	PAS grade I (*n* = 2)	PAS grades II–III (*n* = 3)	No PAS (*n* = 5)
Before training, *n* (%)			
PAS diagnosed	1.75 (87.5)	3.00 (100)	3.19 (63.8)
No PAS diagnosed	0.25 (12.5)	0 (0)	1.81 (36.2)
After training, *n* (%)			
PAS diagnosed	1.81 (91.5)	3.00 (100)	2.31 (46.3)
No PAS diagnosed	0.19 (9.5)	0 (0)	2.69 (53.7)

The diagnostic performance of each trainee before and after the training program is displayed in Table 3. Twelve of sixteen trainees (75%) demonstrated significant improvement in the detection of PAS using standardized ultrasound markers based on EW‐AIP criteria, while four out of 16 (25%) trainees found no improved skills in the detection of PAS. There was no statistically significant difference in baseline characteristics, including prior ultrasound experience, between trainees with improved post‐training performance and those without improvement (Table 1).

**TABLE 3:** Diagnostic performance of trainees before and after the training program.

Trainee	Before training			After training		
	Sensitivity (%)	Specificity (%)	Accuracy (%)	Sensitivity (%)	Specificity (%)	Accuracy (%)
1	100	0	50	100	20	60
2	100	60	80	100	80	90
3	100	40	70	100	60	80
4	100	40	70	100	40	70
5	100	60	80	100	20	60
6	100	80	90	100	40	70
7	100	20	60	100	80	90
8	100	20	60	100	40	70
9	80	40	60	80	60	70
10	100	40	70	80	40	60
11	80	0	40	100	40	70
12	80	40	60	100	60	80
13	100	20	60	80	60	70
14	100	40	70	100	60	80
15	80	60	70	100	100	100
16	100	20	60	100	60	80
Overall (mean ± SD)	95.0 ± 9.0	36.5 ± 22.2	65.6 ± 12.1	96.3 ± 8.0	53.8 ± 21.6	75.0 ± 11.5

Abbreviation: SD, standard deviation.

As shown in Table 4, addressing the primary objective, the training program was associated with a significant improvement in overall prenatal ultrasound diagnostic performance for PAS. Overall diagnostic accuracy increased by 9.4%, from 65.6 ± 12.1% before training to 75.0 ± 11.5% after training (mean difference, post–pre, 9.38%; 95% CI, 1.0–17.7). Diagnostic specificity also increased significantly by 17.5%, from 36.3 ± 22.2% to 53.8 ± 21.6% following training (mean difference, 17.5%; 95% CI, 3.0–32.0). Diagnostic sensitivity showed a small, non‐significant increase from 95.0 ± 9.0% to 96.3 ± 8.0% after training (mean difference, 1.3%; 95% CI, −4.9 to 7.4). In addition, positive predictive value (PPV) increased from 59.8% (95% CI, 51.0–68.0) before training to 67.5% (95% CI, 58.5–75.5) after training, while the negative predictive value (NPV) improved from 87.9% (95% CI, 72.7–95.2) to 93.5% (95% CI, 82.5–97.8). These findings indicate improved ability to correctly confirm and exclude PAS cases, further supporting the effectiveness of the training program, particularly in enhancing diagnostic specificity.

**TABLE 4:** Overall diagnostic performance of trainees before and after the training program.

Parameter	Before training (% ± SD)	After training (% ± SD)	Mean difference (95% CI)	*p*
Accuracy	65.6 ± 12.1	75.0 ± 11.5	9.38 (1.0 to 17.7)	0.030
Sensitivity	95.0 ± 9.0	96.3 ± 8.0	1.25 (−4.9 to 7.4)	0.669
Specificity	36.3 ± 22.2	53.8 ± 21.6	17.5 (3.0 to 32.0)	0.029
PPV	59.8 (51.0–68.0)	67.5 (58.5–75.5)	–	–
NPV	87.9 (72.7–95.2)	93.5 (82.5–97.8)	–	–

Abbreviations: CI, confidence interval; NPV, negative predictive value; PPV, positive predictive value; SD, standard deviation.

According to Table 5, trainees demonstrated improved detection rates for all evaluated ultrasound markers of PAS, with the exception of focal exophytic mass. For two‐dimensional grayscale ultrasound markers, statistically significant improvements were observed in the detection of loss of the clear zone (56.3 ± 20.3% vs. 70.0 ± 12.6%; mean difference, 13.75%; 95% CI, 1.6–25.9; *P* = 0.029), myometrial thinning (60.0 ± 16.3% vs. 73.0 ± 8.9%; mean difference, 13.75%; 95% CI, 5.8–21.7; *P* = 0.002), and bladder wall interruption (51.9 ± 16.8% vs. 70.0 ± 15.1%; mean difference, 18.13%; 95% CI, 10.5–25.7; *P* < 0.001).

**TABLE 5:** Overall accuracy of ultrasonographic signs of placenta accreta spectrum before and after the training program.

Ultrasonographic sign	Before training (% ± SD)	After training (% ± SD)	Mean difference (%) (95% CI)	*p*
2D grayscale				
Placental bulging	58.1 ± 19.7	60.0 ± 8.9	1.88 (−8.82 to 12.57)	0.714
Abnormal placental lacunae	73.8 ± 12.6	76.9 ± 14.5	3.13 (−8.50 to 14.75)	0.575
Loss of clear zone	56.3 ± 20.3	70.0 ± 12.6	13.75 (1.62 to 25.88)	0.029
Myometrial thinning	60.0 ± 16.3	73.8 ± 8.9	13.75 (5.76 to 21.74)	0.002
Bladder wall interruption	51.9 ± 16.8	70.0 ± 15.1	18.13 (10.53 to 25.72)	< 0.001
Focal exophytic mass	N/A	N/A	N/A	N/A
Color Doppler				
Uterovesical hypervascularity	60.0 ± 15.5	71.9 ± 12.2	11.88 (4.28 to 19.47)	0.005
Bridging vessels	56.9 ± 15.4	65.0 ± 11.0	8.13 (−0.84 to 17.09)	0.072
Subplacental hypervascularity	55.0 ± 22.0	68.1 ± 18.7	13.13 (−0.87 to 27.12)	0.064
Placental lacunae feeder vessels	61.3 ± 20.6	73.1 ± 14.5	11.86 (0.66 to 23.09)	0.039

Abbreviations: 2D, two‐dimensional; CI, confidence interval; N/A, not applicable; SD, standard deviation.

For color Doppler ultrasound markers, significant improvements were noted in the detection of uterovesical hypervascularity (60.0 ± 15.5% vs. 71.9 ± 12.2%; mean difference, 11.88%; 95% CI, 4.3–19.5; *P* = 0.005) and placental lacunae feeder vessels (61.3 ± 20.6% vs. 73.1 ± 14.5%; mean difference, 11.86%; 95% CI, 0.7–23.1; *P* = 0.039).

The ultrasound marker of focal exophytic mass was not included in the pre‐ and post‐training assessments. This marker is almost exclusively observed in the most invasive forms of PAS and is reported in approximately 10% of cases, typically during the third trimester.^19^ Owing to its rarity, limited representative data were available, and only a single illustrative video clip from our center was included in the training lecture.

The learners self‐assessed that they had improved knowledge and understanding of PAS and PAS ultrasonography markers according to the post‐training satisfaction assessment (Table 6). Most trainees (81.25%) gave their prior understanding of PAS a fair to average rating at the beginning of the program. Following training, 68.75% of participants regarded their understanding of PAS as good, while 25% rated it as ordinary. All trainees expressed satisfaction with the curriculum and a higher level of confidence in their ability to diagnose PAS using ultrasound markers.

**TABLE 6:** Post‐training satisfaction survey.

Characteristic	Overall (*n* = 16)	Improved PAS detection (*n* = 12)	No improvement (*n* = 4)	*p*
Knowledge and understanding of U/S PAS markers, *n* (%)				0.195
Excellent	1 (6.25)	0 (0)	1 (25)	
Good	11 (68.75)	9 (75)	2 (50)	
Average	4 (25)	3 (25)	1 (25)	
Fair	0 (0)	0 (0)	0 (0)	
Poor	0 (0)	0 (0)	0 (0)	
Level of confidence in using U/S markers for PAS diagnosis				N/A
High confidence	0 (0)	0 (0)	0 (0)	
Confidence	8 (50)	6 (50)	2 (50)	
Some confidence	8 (50)	6 (50)	2 (50)	
Little confidence	0 (0)	0 (0)	0 (0)	
No confidence	0 (0)	0 (0)	0 (0)	
Level of satisfaction about this program				N/A
Very satisfied	9 (56.25)	7 (58.33)	2 (50)	
Satisfied	7 (43.75)	5 (41.67)	2 (50)	
Unsure	0 (0)	0 (0)	0 (0)	
Dissatisfied	0 (0)	0 (0)	0 (0)	
Very dissatisfied	0 (0)	0 (0)	0 (0)	
Number of PAS cases detected in practice after training program				0.862
0	8 (50)	6 (50)	2 (50)	
1	6 (37.5)	4 (33.33)	2 (50)	
2	1 (6.25)	1 (8.33)	0 (0)	
≥ 3	1 (6.25)	1 (8.33)	0 (0)	

*Note*: Data are presented as mean ± standard deviation (SD), number (%), or number (min–max).

Abbreviations: PAS, placenta accreta spectrum; TAS, transabdominal ultrasound; U/S, ultrasound.

## 4 DISCUSSION

Accurate prenatal diagnosis is fundamental to reduce the burden of maternal and fetal morbidity and mortality associated with PAS by allowing pre‐planned treatment in excellent centers with high experience in surgical management.^10^, ^22^ Recent studies have increasingly applied machine learning and deep learning techniques to improve diagnostic accuracy across a wide range of medical conditions, particularly in image‐based classification tasks such as cancer detection.^24^, ^25^ While these approaches demonstrate strong performance, their implementation in routine obstetric ultrasound practice remains limited. In contrast, our study focuses on improving human diagnostic performance through a structured educational approach. While not algorithm driven, this method offers a practical and scalable solution that can be readily implemented without the need for advanced computational systems. Importantly, it complements emerging artificial intelligence approaches by strengthening the foundational skills required for accurate ultrasound interpretation.

Overall strategies to improve the prenatal diagnosis of PAS require comprehensive knowledge of the pathophysiology of PAS, identification of women at risk, and improvement in ultrasound equipment and operator skills.^7^ The use of standardized ultrasonographic signs of PAS plays an important role in improving prenatal diagnostic performance, particularly specificity.^7^ In this prospective educational study, we demonstrated that a structured, video‐based ultrasound training program significantly improved overall diagnostic accuracy and specificity for PAS among trainees. The incorporation of dynamic ultrasound video clips, rather than static images, provides a more realistic simulation of clinical practice and might enhance pattern‐recognition skills required for real‐time diagnosis. Participants showed enhanced recognition of grayscale and color Doppler markers and greater confidence in differentiating PAS from non‐PAS cases.

From an efficiency perspective, the training program required relatively short, structured instructional time (approximately 135 min per participant) and leveraged pre‐existing ultrasound video databases and widely accessible online platforms. No additional memory‐intensive computation or specialized hardware was required, highlighting the feasibility and scalability of this approach, particularly in resource‐limited settings. The observed improvement in specificity is particularly important, as overdiagnosis might lead to unnecessary referral, patient anxiety, and resource utilization.

Our findings are consistent with previous educational interventions,^20^, ^21^ which demonstrated improved diagnostic performance following structured PAS training. However, several key differences between these studies and the present study should be highlighted.

In terms of methodology, prior studies [19. 20] primarily used static ultrasound images, whereas our study employed dynamic ultrasound video clips. This distinction is important because ultrasonography is inherently a real‐time modality; video‐based assessment better reflects clinical practice but requires more advanced interpretation skills, including continuous spatial and temporal evaluation.

Regarding outcomes, prior study reported a greater improvement in diagnostic accuracy (approximately 17.3%),^20^ whereas our study demonstrated a more modest increase of 9.4%. This difference is likely due to the higher complexity of interpreting dynamic video clips compared with static images.

Our study demonstrated a substantial improvement in diagnostic specificity (17.5%), which is particularly clinically relevant. Improved specificity reduces false‐positive diagnoses, thereby minimizing unnecessary referrals, patient anxiety, and healthcare resource utilization. In contrast, previous studies have focused more on overall accuracy or sensitivity, with less emphasis on specificity.

Finally, our dataset consisted of histopathologically confirmed PAS cases presented in video format, ensuring high diagnostic validity and enhancing the realism of the training material. Overall, although the improvement in accuracy was smaller than in prior reports, the use of dynamic imaging and the significant gain in specificity suggest that our training model provides a more clinically realistic and practically applicable approach to improving PAS diagnosis.

While ablation studies and cross‐validation are not applicable to this educational intervention, several design elements were incorporated to enhance robustness and reduce potential bias. These included expert validation of training materials, use of standardized diagnostic criteria, randomized ordering of assessment cases, and consistent evaluation conditions between pretest and posttest. Together, these measures help mitigate overfitting to specific cases and support the reliability of the observed improvements in diagnostic performance.

In routine practice, integration of clinical risk factors with imaging findings would likely further enhance diagnostic performance beyond that observed in this controlled setting. The trainee population—third‐year residents with baseline ultrasound competency—represents general obstetric providers who are often responsible for initial PAS screening, thereby supporting the practical relevance of this intervention.

Some limitations should be acknowledged. First, the sample size was modest and derived from a single institution, which might limit generalizability. Second, individual variability in learning ability and baseline proficiency (propensity) was not formally analyzed, which might influence the degree of improvement observed among trainees. Third, certain advanced markers, such as focal exophytic mass associated with severe PAS (FIGO grade IIIC), were underrepresented due to limited case availability. Fourth, participants were aware that the training aimed to improve PAS diagnosis, which might have introduced expectation bias and contributed to high baseline sensitivity through oversuspicion. In addition, the use of a fixed set of video cases for both pretest and posttest assessments, although presented in randomized order, might introduce a degree of recall bias. Although variability among trainees was observed (reflected by relatively wide standard deviations), statistically significant improvements in accuracy and specificity indicate a consistent overall benefit of the training program across participants. Finally, clinical information and patient risk factors were intentionally withheld to isolate ultrasound interpretation skills; however, this might not fully reflect real‐world diagnostic conditions where clinical context contributes to decision‐making.

In summary, this structured, video‐based PAS ultrasound training program significantly improved diagnostic performance among trainees. Incorporation of dynamic imaging and standardized ultrasound markers offers a practical framework for strengthening prenatal PAS detection.

From a cost perspective, the training program was highly resource efficient. It utilized existing institutional ultrasound equipment and archived video databases, thereby avoiding the need for additional imaging acquisition costs. The educational intervention was delivered through standard lecture formats and widely available online platforms (e.g., web‐based video access and form‐based assessment tools), minimizing software expenses. No specialized hardware or proprietary software was required. Therefore, the overall implementation cost is low, supporting feasibility and scalability in both high‐ and low‐resource settings.

Expansion of this model into broader, multicenter training initiatives might contribute to improved early recognition and referral, ultimately enhancing maternal outcomes.

Further research with larger, multicenter datasets, particularly including advanced invasive markers, is warranted to optimize training strategies and improve the generalizability of the findings.

## 5 CONCLUSION

Participation in a structured, video‐based ultrasound training program was associated with a significant improvement in prenatal diagnostic accuracy for PAS, with a 9.4% increase in overall accuracy and a 17.5% improvement in specificity, while maintaining high sensitivity. Trainees demonstrated enhanced recognition of standardized PAS ultrasound markers and improved diagnostic performance. These findings are clinically important, as increased specificity might reduce false‐positive diagnoses, unnecessary referrals, and associated healthcare burden. Broader implementation of this training model might enhance early detection and facilitate timely referral to tertiary centers, with the potential to improve maternal outcomes. Future studies should include multicenter validation, greater case diversity, and integration of simulation‐based and technology‐assisted training approaches to further improve diagnostic performance.

## AUTHOR CONTRIBUTIONS

Nicha Vetvitayavatana conceived and designed the study, collected and analyzed the data, performed statistical analysis, and drafted the manuscript. Julaporn Pooliam calculated the sample size, contributed to data analysis, data interpretation, and statistical analysis and provided expert advice on statistical methodology. Pharuhas Chanprapaph supervised the study and contributed to study design, data interpretation, statistical analysis, and critical revision of the manuscript for important intellectual content. All authors have read and approved the final version of the manuscript and agree to be accountable for all aspects of the work.

## FUNDING INFORMATION

This research project has no financial support.

## CONFLICT OF INTEREST STATEMENT

The authors hereby declared no personal or professional conflicts of interest regarding any aspect of this study.

## ETHICS STATEMENT

This study was approved by the Siriraj Institutional Review Board, Faculty of Medicine Siriraj Hospital, Mahidol University (IRB no. SI 278/2022). Written informed consent was obtained from all participants.

## Supporting information


**Figure S1.** Algorithm: Structured Video‐Based PAS Ultrasound Training.

**Table S1.** Dataset and Statistical Analysis Summary.

## Data Availability

Research data are not shared.
