# Supplementary material for: The effectiveness of a training program in improving the prenatal diagnosis accuracy of placenta accreta spectrum with ultrasound
Source: Int J Gynaecol Obstet. 2026 Jun 27;175(3):1405–15. doi: 10.1002/ijgo.71127 (PMC13769365; doi:10.1002/ijgo.71127)
Supplement: Supplementary file 1 [file IJGO-175-1405-s001.docx]

**Table S1. Dataset and Statistical Analysis Summary**

| **Category** | **Parameter** | **Description** |
| --- | --- | --- |
| **Dataset** | Cases per test | 10 (5 PAS: 2 Grade I, 3 Grade II–III; 5 non-PAS) |
|  | Source & validation | Histopathology-confirmed; reviewed by 3 MFM experts |
|  | Test design | Same cases in pre/posttest (reordered) |
|  | Video characteristics | 4 min/case; 3–4 loops |
| **Analysis** | Software | SPSS v21 |
|  | Data presentation | Mean ± SD / median / % |
|  | Statistical tests | Paired t-test or Wilcoxon signed-rank test |
|  | Significance & CI | p < 0.05; 95% CI |
|  | Performance metrics | Accuracy, sensitivity, specificity |
