# Supplementary material for: The effectiveness of a training program in improving the prenatal diagnosis accuracy of placenta accreta spectrum with ultrasound
Source: Int J Gynaecol Obstet. 2026 Jun 27;175(3):1405–15. doi: 10.1002/ijgo.71127 (PMC13769365; doi:10.1002/ijgo.71127)
Supplement: Supplementary file 2 [file IJGO-175-1405-s002.docx]

**Supplementary Figure S1. Algorithm: Structured Video-Based PAS Ultrasound Training**

1. **Input:** Eligible OB/GYN trainees with basic ultrasound experience
2. **Obtain consent and provide orientation**
3. **Pretest:** (45-minute)
   - Assess 10 video cases (5 PAS, 5 non-PAS)
   - Record marker identification and diagnosis
4. **Training:**
   - Deliver 40-minute structured lecture
   - Provide interactive case practice
   - Allow 2-month access to recorded materials
5. **Posttest:** (45-minute)
   - Reassess same cases in different order
   - Record responses
6. **Output:**
   - Compare pre- and posttest performance (accuracy, sensitivity, specificity)
7. **Perform statistical analysis**

The total structured training time was approximately 135 minutes, using existing institutional resources without additional hardware requirements.
